# Integrating Turkish Work and Achievement Goals With Schwartz’s Human Values

**DOI:** 10.5964/ejop.v11i2.825

**Published:** 2015-05-29

**Authors:** Suna Tevrüz, Tülay Turgut, Murat Çinko

**Affiliations:** a Division of Organizational Behavior, Department of Business Administration, Marmara University, Istanbul, Turkey; b Division of Quantitative Methods, Department of Business Administration, Marmara University, Istanbul, Turkey; Academy of Special Education, Warsaw, Poland

**Keywords:** basic human values, emic studies, work goal, achievement goal, intrinsic motivation

## Abstract

The purpose of this study was to examine the integration of indigenous values developed in Turkey to Schwartz’s universal values. Students (N = 593) from six universities in Istanbul responded the value scale, which consists of 10 etic PVQ items (each item representing one of 10 main Schwartz values) and 23 emic WAG items (representing work-achievement goals). PROXSCAL, a multidimensional scaling method, was used to test whether etic and emic sets of values integrate and form the universal circular structure proposed in Schwartz value theory. The motivational continuum of values as a circular structure was similar to pan-cultural results, but adding another value type to the openness to change pole. While some of the items in this region represent autonomy of thought, remaining items diverge. The principle of conflicting values on opposite poles was not supported in relation to openness to change-conservation dimension. These two poles had similar priorities, contrasting with pan-cultural results, and demonstrating a culture-specific aspect of responding to motivational goals. Insights gained by emic studies will be functional in enriching understanding values, and contributing to the comprehensiveness and universality of Schwartz value theory.

Values are among the various influences affecting human behavior. They function as a guiding force, shaping our attitudes, and behaviors. Although for decades, philosophers and social scientists were interested in values, no theory as comprehensive and integrative as Schwartz’s emerged. Influenced by Kluckhohn’s (1949; Kluckhohn & Strodtbeck, 1961) and Rokeach’s (1973) preeminent studies, and getting impetus from Hofstede’s (1984) world-wide work, Shalom H. Schwartz developed his prevalent theory (Schwartz & Bilsky, 1987, 1990). The theory proposes the main human values as well as the underlying value structure as universals. With an etic approach it assumes that different cultures can be compared on generalizable grounds. This encourages researchers to test the goodness of fit of indigenous research data to Schwartz’s universal values.

The aim of this study is to search the degree of fit between an indigenous value research and Schwartz’s universal values. First, let’s have a brief look at Schwartz value theory.

Majority of Schwartz’s studies are directed towards the universal structure of values. He makes distinctions between individual and cultural level studies stating that, the dimensions underlying values at different levels are not the same. Individual and cultural level approaches are independent from each other, because value priorities may show difference in two levels. At the individual level a person may give importance to self-direction, but at the cultural level the emphasized value may be obeying to authority (Smith & Schwartz, 1997). Therefore, the dimensions he proposes for two levels are different. Since the focus of the present study is on the individual, we’ll go over Schwartz’s theory from the individual level perspective.

Individual-level values originate from universal requirements of human existence, which are 1) biological needs, 2) requisites of coordinated social interaction, and 3) demands for group functioning and survival. These requirements are represented cognitively in the form of values as conscious goals or values (Schwartz & Bilsky, 1987, 1990; Smith & Schwartz, 1997). Sexual needs, for instance, may be transformed into intimacy or love values; need for coordinated social interaction may show itself as equality or honesty values; group survival may be represented as values for national security or world peace (Schwartz & Bilsky, 1987).

Schwartz defines values as; *conceptions of the desirable that guide the way* social *actors (e.g. organizational leaders, policy makers, individual persons) select actions, evaluate people and events and explain their actions and evaluations. They are trans-situational criteria or goal, ordered by importance as guiding principles in life* (Schwartz, 1999, pp. 24-25). In this definition Kluckhohn’s and Rokeach’s views are combined, with the addition of the concept, “goal”. In his theory the concept goal is important because, it is the motivational goal, which distinguishes value types.

Distinct types of values were derived from the three individual-level universal requirements. From various sources (including world’s major religions, surveys developed in various countries, and Rokeach values) 10 groups of values with distinct motivational goals are drawn. These are: Power, Achievement, Hedonism, Stimulation, Self-direction, Universalism, Benevolence, Tradition, Conformity and Security (Schwartz & Bilsky, 1987, 1990; Schwartz, 1992, 2012). In an earlier study spiritualism was included as the 11th value, but lacking consistency in meaning across cultures, it is not used. The above-mentioned ten value types are the basic human values of people in any culture (Schwartz, 2012), related in a circular structure. It seems as if a basic principle is at work in ordering the values on a motivational continuum. In their self-determination theory, Ryan and Deci (2000) also propose motivation types, which are ordered on a continuum from controlled to autonomous regulation. Introjected regulation seems to work for the motivating role of power and achievement, which have extrinsic contingencies like tangible rewards and evaluative comparisons by social standards. Values at the conservation pole seem to fit motivation with identified regulation. We may not personally own them, but our acceptance of them as important may motivate us. When identified regulations are assimilated to the self, the regulation becomes more autonomous, and motivation with integrated regulation occurs (Gagné & Deci, 2005; Ryan & Deci, 2000). Benevolence and universalism seem to fit motivation by integrated regulation, since performing these values needs to assimilate and own them as personally important, although the activities themselves may not be so attractive. Full autonomy or self-regulation comes with intrinsic motivation. Individuals are intrinsically motivated when they derive satisfaction from the activity itself. Intrinsically motivated individual wants to extend and use capacities, to look for novelties and challenges, to learn and to master. The underlying needs are autonomy and competence; values on the openness to change pole are such values. Ryan and Deci (2000, p. 70) state that, “perhaps no single phenomenon reflects the positive potential of human nature as much as intrinsic motivation”.

The circular arrangement of values represents a motivational continuum. The closer the values around the circle, the more similar their motivational goal is; so the correlation between them is more positive. The more distant they are, the more dissimilar they are consequently thus more negative the relationship. This means, when finding a certain relation between a specific value and some other variable, same relation with similar value types may be expected (Schwartz, 2012).

These ten value types are organized in two bipolar dimensions: 1) Openness to change vs. Conservation and 2) Self-enhancement vs. Self-transcendence. However, there are studies indicating that only eight out of 10 values reflect these polarized dimensions; the two peripheral values are tradition and hedonism (Welzel, 2010).

The most important aspect of the theory is the structure of values, which is formed by dynamic relations between value types (Schwartz, 2012). The motivational goal of some values conflict and some are congruent with each other. For instance, universalism conflicts with hedonism while benevolence is congruent with universalism. Conflicting-Congruent relations create a continuum of related values in a circular structure (Figure 1).

**Figure 1:**
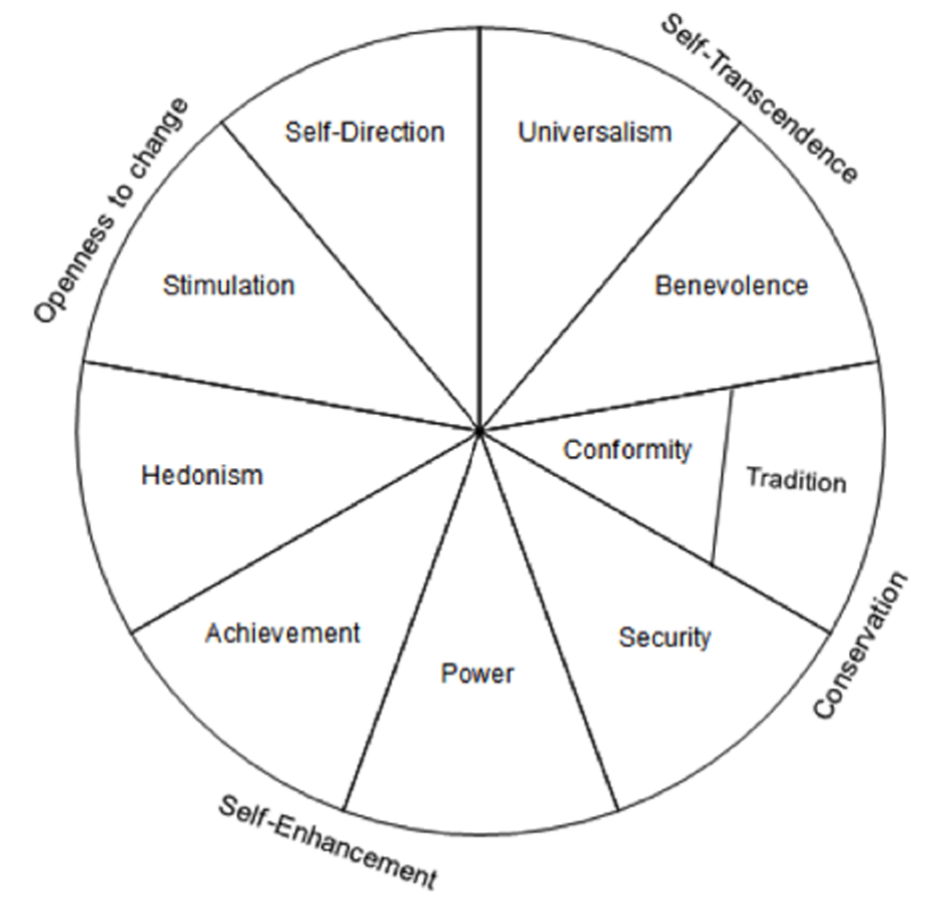
Schwartz’s theoretical model of ten values (Schwartz & Sagie, 2000).

Besides congruence and conflict, Schwartz (2012) suggests two other organizing principles. One being the interest values serve. Do they serve self-interest, or the interest for others? For instance benevolence-universalism-tradition-conformity is concerned with interest for others. These values form the social focus pole of this dimension. Power-achievement-hedonism-stimulation-self direction on the other hand is concerned with personal interests, forming the pole of personal focus. This interest dimension resembles Hofstede’s individualism/collectivism dimension. The second suggested principle is the relation of values to anxiety. Some values serve to cope with anxiety while some others are free of anxiety. Anxiety based values are also termed as *prevention of loss goal* and *self-protection against threat*. Alternative terms used for anxiety-free values are: *Promotion of gain goals* and *self-expansion and growth* (Schwartz, 2012). Anxiety based-Anxiety free dimension calls to mind promotion-prevention concepts in Higgins’ (1998) self-regulation theory. It also resembles principles of positive-negative reinforcement. Schwartz (2012) outlines these three value-structuring principles as in Figure 2.

**Figure 2:**
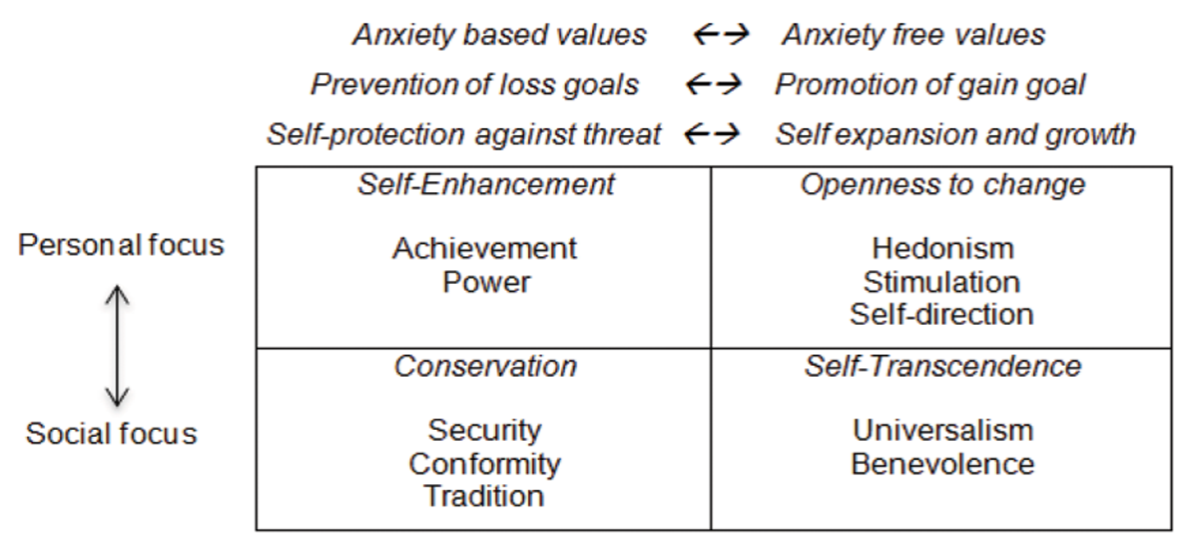
Organizing principles of the value structure (Schwartz, 2012).

Schwartz’s first instrument to measure values is known as Schwartz Value Survey (SVS) including 56 or 57 items. Like Rokeach Value Survey (RVS), some items are nouns of desirable end-states, and some are verbs of desirable ways of acting. Respondents rate each value item as a “guiding principle in his/her life”. Both in individual and culture level studies, multidimensional scaling analysis are used for the organization of values into dimensions. Findings of individual level studies gave the same structure in samples of 67 nations. However, some studies done with SVS in samples from less developed nations do not support the theory (Schwartz, 2012).

Reasoning, that evaluation of specific value items in SVS need abstract thinking, an alternative instrument, a more concrete one was developed known as Portrait Value Questionnaire (PVQ) (Schwartz, 2012; Schwartz et al., 2001). PVQ measures values indirectly through judgments of similarity. The original PVQ consists of 40 items describing 40 different people. Each item describes the goal, aspirations or wishes important for that person. Each description implicitly points to a value. For each item, respondents answer, “how much like you is this person?”

PVQ with 29 items was applied to populations in which the theory was not supported with SVS (Black South African university students and Ugandan teachers). It was also administered to a national sample of a developed country (Italians) where the theorized structure was confirmed by SVS (Schwartz et al., 2001). Data collected from all these sources supported the theorized structure, indicating that PVQ can measure the ten values (Schwartz et al., 2001). European Social Survey used the 21-item short version of PVQ and attained the same result (Schwartz, 2012). In world value survey a shorter version (10-items) was used (Welzel, 2010).

The theory proposes the main human values as well as the value structure as universals. With an etic approach it assumes that different cultures can be compared on generalizable grounds. This encourages researchers to test the interlocking of indigenous value research data in Schwartz’s universal values. This is exactly one of the objectives of the emic value survey applied to university students in Turkey, the measuring device of which was developed through a series of studies. Before going into the present study, development of the value scale is briefly explained.

## Development of the Value Scale With Turkish University Students

Value scale construction passed through several preliminary research studies beginning with two open-ended questions, one for work-goal^i^ and the other for achievement-goal.

Question for work-goal was: “What do you think is the purpose for working? Why do people work?” Respondents were 239 university students and working people (Tevrüz & Turgut, 2004). After eliminating synonymous, overlapping and unrepresentative responses, the remaining 79 work-goal terms were itemized and applied to 417 undergraduate university students and 438 adults from different professions. They rated the importance they give to the work-goals on a 6-point agree-disagree scale (Tevrüz & Turgut, 2004; Turgut & Tevrüz, 2003).

The open-ended question for achievement-goal was: “What does being successful mean? Which achievement of yours you consider as a success?” Respondents were 71 university students and their parents (*N* = 142) (Tevrüz, 1995). After eliminating synonymous, overlapping and unrepresentative responses, the remaining 75 achievement-goal terms were itemized and applied to 576 university students. Instruction for the achievement-goal scale was: “Assume that you have achieved the situations listed below how much would you say you really feel successful on each situation?” Response scales ranged from 1 (*none at all*) to 6 (*very much*)^ii^.

Factor analyses for work-goal gave 12 (62% variance), and achievement-goal gave 11 orthogonal factors (63% variance). Second-step factor analysis for each study distributed the factors into three groups. As can be seen on Table 1 factors of both concepts are grouped in similar dimensions: 1) normative values, 2) enrichment values, and 3) worldly values. This result of second order factor analysis somewhat demonstrated the three functions values play in our lives as individuals. Although taken from the individuals’ perspective, these functions call to mind Schwartz’s universal requirements. Worldly values remind biological needs; social or normative values meet coordinated social interaction and group functioning. On the other hand, intrinsic values seem to rise from a need of self-enrichment, which has no direct correspondence with Schwartz’s universal requirements.

**Table 1:** Factors and Dimensions of Achievement-Goal and Work-Goal

Achievement-Goal	Work-Goal
Social/Normative values	
To be trustworthy To set up a proper family To make arrangements for children’s future To be happy^a^	To contribute to society To avoid missteps To fulfill religious duties To set order in one’s life
Enrichment/ Growth values	
To go beyond one’s capacity To have integrity To have enthusiasm To be creative To protect environment	To perform the desired profession^b^ To be enriched in knowledge and to use it To have a meaningful life To have an active life To have a pleasant life To be busy
Worldly/ Extrinsic values	
To obtain position in society To gain money	To ensure livelihood^c^ To gain status

^a^Happiness is excluded in the present study. ^b^Students in Turkey cannot directly choose the graduate program they prefer. Instead, they make 25-30 choices and are expected to comply with the one, which coincides with their exam-score. Mostly this is not what they mostly prefer. So, performing the desired profession becomes an important goal and motivation for university students. ^c^“To ensure livelihood” is reconstructed as two items in this study: 1. Family wellbeing, 2. Livelihood.

The similarity of dimensions made the authors assume that, these two constructs are interrelated in that, desire for goal includes a desire for success and that desire for success requires working for a goal. A study done with the reconstructed shortened form of the two scales supported the overlapping between the two constructs (Tevrüz, Turgut, & Çinko, 2010). 1183 university students (undergraduate and graduate) from different regions of Turkey participated. Achievement goal was measured with 11 items, representing 11 factors. Each item was a description of a person which resembled PVQ items. The descriptions were formed with the achievement goal responses, which constituted the given factor. Participants were asked to evaluate how important the described person’s success was and in whose place s/he would want to be. Work goal scale was also reconstructed with the same procedure. Participants rated how important it was to achieve the described work goal, and for which goal s/he was studying as a university student. Items, of each concept were grouped in three factors. The high correlation and regression values between the two groups supported the overlapping nature of the two constructs (Tevrüz et al., 2010).

It seems in whichever content the goals are identified, their motivational function takes the same route. One of the routes takes the person to intrinsic or self-enriching values; another to extrinsic or worldly values; and the third one to social/normative values. Combination of these two goal-sets gives an integrated value scale enriched in item variety, which we called WAG (Work-Achievement Goal) scale. This is the result of an emic study, using the method of factor analysis, which is different from the multidimensional scaling method (MDS) used for SVS and PVQ. However, the above-mentioned three dimensions remind the dimensions of Schwartz’s universal main values. Self-enriching goals seem to reflect openness to change. Worldly goals seem to reflect self-enhancement. Social/Normative goals resemble values of conservation. Although there seems to have vacancy in WAG values for the self-transcendence dimension, some emic values, given on Table 1, such as “to protect environment” and “to contribute to society” may take place at self-transcendence dimension when analyzed with MDS.

The observation of nearly similar dimensions of an emic and the etic value studies made us to ask and inquire where these emic values take place in the etic value structure when analyzed with MDS. So, whether the emic dimensions and the value types correspond with the motivational structure of Schwartz’s value model was the main question of this present study.

## Method

### Participants and Procedure

A total of 593 undergraduate and graduate students from six universities in Istanbul (348 females, 245 males) participated. Students were invited to participate on voluntary basis, and asked to complete the questionnaire in class hours. It was emphasized that the participants should not write their names or any identifying marks on the questionnaire.

### Instrument

WAG consists of 23 items. Ten of them are from orthogonal achievement goal factors, each one describing a person with the goal-items constituting that specific factor. Twelve work goal items are developed with the same procedure. However, the description terms of the factor “to ensure livelihood”, which was formed and used in the previous study as a single item was criticized as describing two different conditions: one related to gaining money and the other related to family well-being. So forming a separate descriptive “family wellbeing” item increased work-goal items to 13. Including 10 items representing Schwartz’s 10 main values^iii^ made a total of 33-item value-scale (items are given at Appendix A). 10-items are mainly chosen from a Turkish translation of 40-item PVQ scale (Demirutku, 2007) with minor changes on the Turkish translation. The participants were asked to answer the question, “How similar is this person to you?” Responses were given on a scale ranging from 1 (*not a bit similar to me*) to 6 (*very much similar to me*).

Briefly, the value scale used in this study consists of 10 etic PVQ items (each item representing one of 10 main Schwartz values) and 23 emic WAG items (representing work-achievement goals). The purpose was to see if these two sets of values integrate and form the universal circular structure proposed in Schwartz value theory.

## Results

### Arrangement of WAG Items

**Figure 3:**
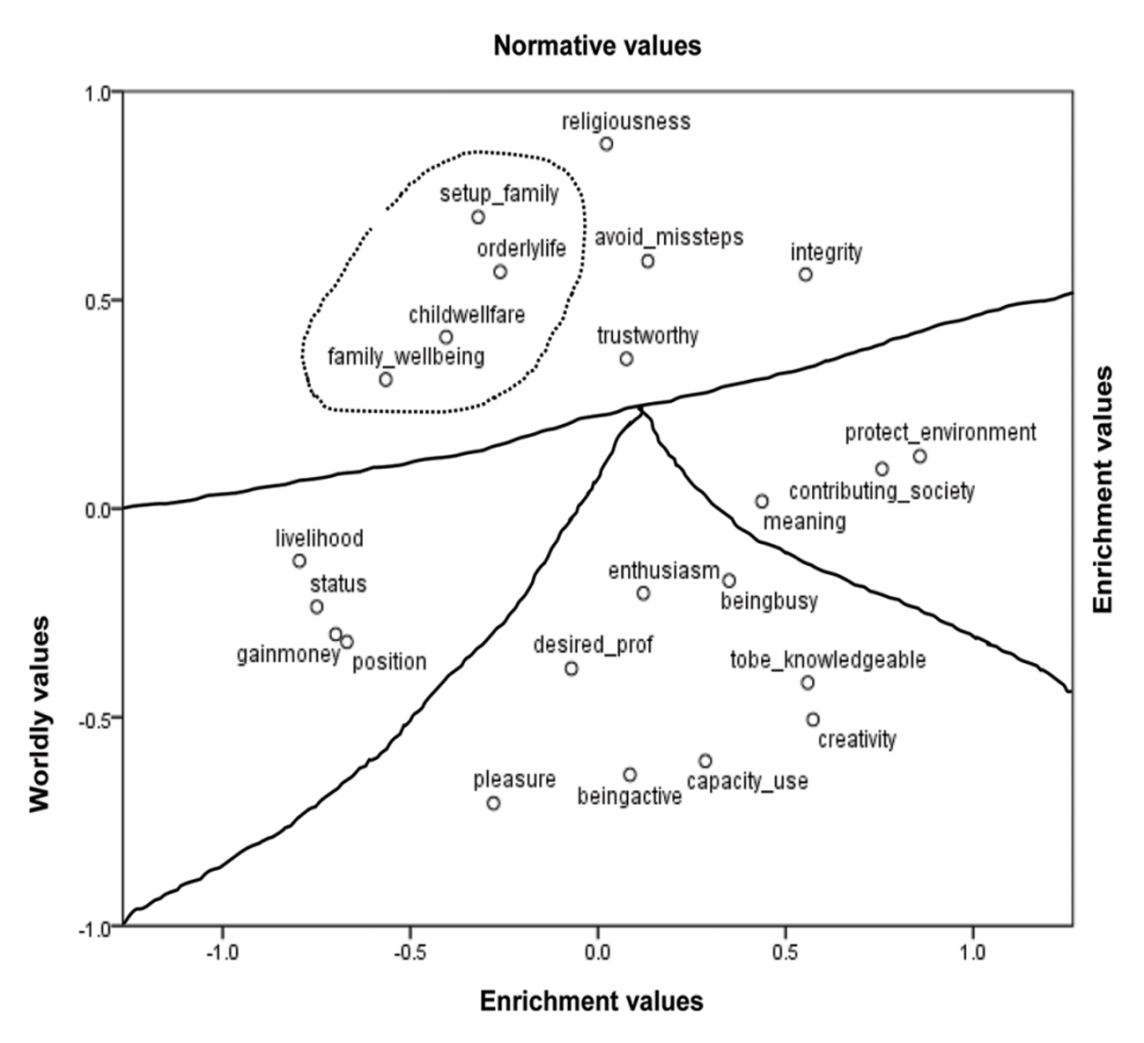
Configuration derived from WAG items.

**Figure 4:**
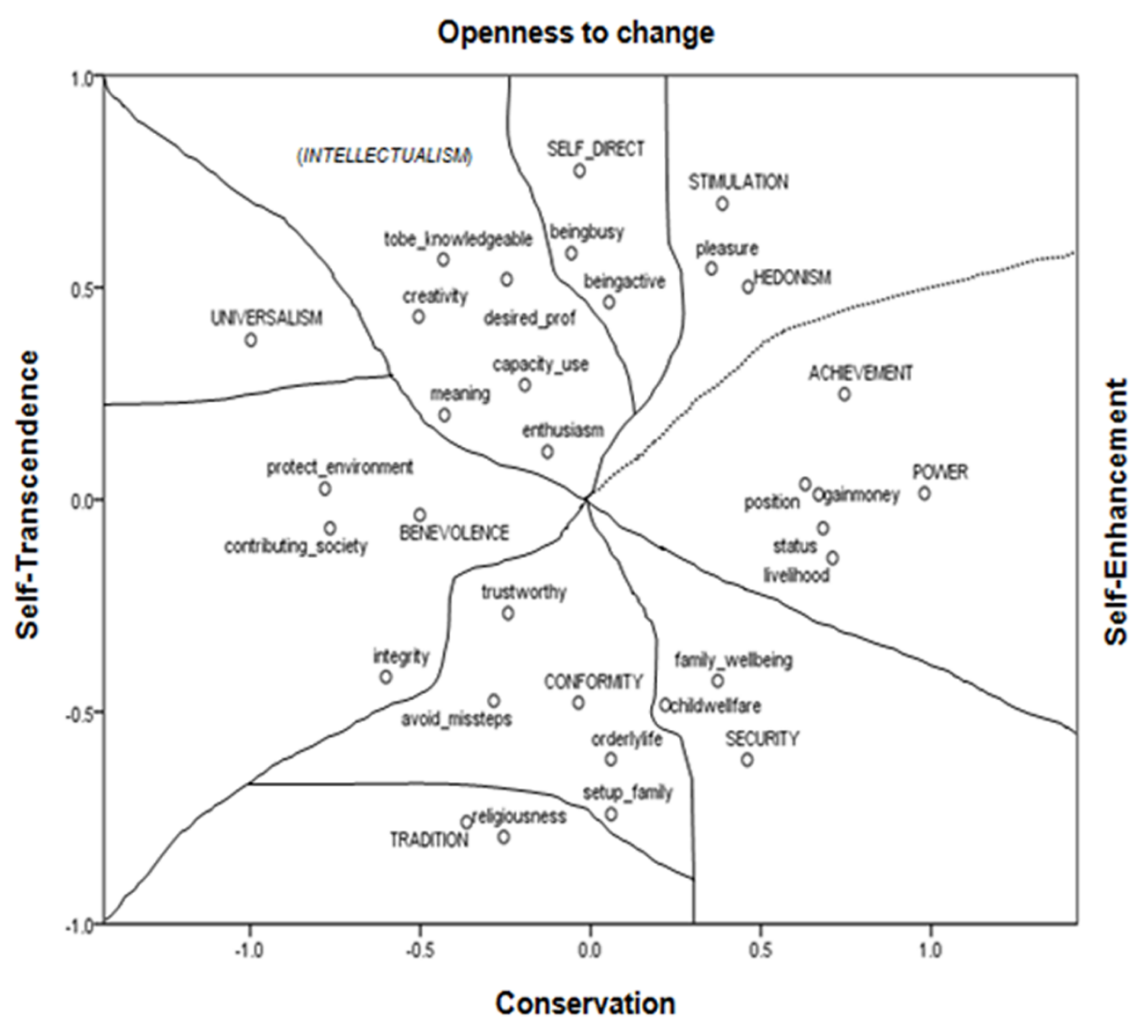
Configuration derived from the combination of WAG and PVQ items (with capital letters).

Using PROXSCAL, which is a multidimensional scaling method in SPSS, we first looked at the spatial configuration of 23 WAG items on the two-dimensionally structured value circle (Figure 3), then analyzed the combination of the two scales with the same procedure (Figure 4). The Pearson correlation coefficients are used as similarity proximities. Transformation proximity is interval and no restriction is given to common space. Simplex is used as an initial starting configuration where stress convergence and minimum stress are 0.0001 and maximum iteration is 100.

The circular structural form repeats itself with the WAG items. The three higher order factors found in work-achievement study (Tevrüz et al., 2010) given at Table 1, are separated to four regions, which call attention to Schwartz’s two bipolar dimensions with four higher order value types. Worldly values correspond to self-enhancement, and normative values to conservation. What was called in the work-achievement goal study as enrichment values seem to be decomposed here as “openness to change” with self-focused values (such as enthusiasm, performing desired profession, being busy, to be knowledgeable, creativity, pleasure, being active, capacity use) and as self-transcendence with other-focused values (such as protect environment, contribute to society, meaningful life).

On the normative region there are eight value items, four of which seem to be slightly separated from the others. These items (set up a family, an orderly life, welfare of children and family wellbeing) come closer together forming a value area related to “family” signifying family as a societal institution. The family wellbeing, and ensuring livelihood items which were rated as a single item in our previous study, took place in different regions in this study. Family wellbeing moved to normative-values region, whereas livelihood preserved its place in the worldly-values region.

### Arrangement of WAG + PVQ Items

Addition of 10 PVQ items into the analysis gave the form in Figure 4. The ordering of the 10 PVQ items (written with capital letters in Figure 4) is according to the motivational goal they express. That is; they take place under the same dimensions and in the same compatible-conflicting order as stated in the theoretical model. WAG items, which form a group within a particular higher order value, have conceptually the same meaning as that higher order value type. So even visually it is possible to discriminate them to four higher order value regions. However discrimination of the emic value items to Schwartz value types is not as clear.

In order to specify concomitant emic value items with Schwartz value types, distances index (given at Appendix B) is used, and the lowest distance score is applied as the discrimination criterion. As seen from Figure 4, 33 items are distributed to nine distinct regions, one of which includes hedonism and stimulation together, both entailing a desire for pleasant arousal and the other, power and achievement together, both emphasizing social superiority and esteem (Schwartz, 1992). Universalism, on the other hand, is isolated; none of the emic values have correspondence with universalism. Universalism, as a value type, denotes contact with outsiders and nature, but the emic items bearing just these meanings gather around benevolence (which denotes contact with close others) rather than universalism. Examination of these items gives some insight into this somewhat inverse grouping. In all the items around benevolence (contribute to society, protect environment and even integrity) and in benevolence itself, the person is in action for others, for society, for environment or with the self (Items 1, 21, 22, 25 in Appendix A). Whereas in the universalism item the person doesn’t do anything, s/he just informs a “should” belief. A universalism item with action terms would have demonstrated a different picture.

On the conservation pole the separation between conformity and security zones was rather arbitrary, because the distance of child welfare from conformity and from security wavers. It is nearer to orderly life (.205), which is under conformity, but its lowest distance is with family wellbeing (.164), which has its nearest distance to security (.205). Moreover, trying to provide a sound future for the off spring is more indicative of securing them than denoting the domination of social expectations. So, child welfare took its place at the security region.

The same problem arose with the location of integrity. Its distance from the values of conformity and benevolence were close (from avoid missteps and trustworthiness was respectively .322 and .389, and from contribute to society and benevolence was .387 and .384) (Appendix B). Although the distance scores were closer to conformity, the item description (“having principles and never giving them up”: Item 22 in Appendix A) seemed to represent a motivation type, which in terms of self-determination theory was more autonomous than conformity values and closer to integrated regulation rather than identified regulation. Moreover as a value it was more anxiety free than the conformity values. So, integrity is located at the benevolence region.

On the openness to change pole, stimulation and hedonism, as a single region, take-in the emic item pleasure with nearly equal distance (.156 for stimulation and .116 for hedonism). Pleasure unites these two value types.

### A New Value Region

Being busy and being active join self-direction. Self-direction signifies autonomy and is defined as independent thought and action (choosing, creating, exploring), and is derived from organismic needs for control and mastery (Schwartz, 1992, 2012). Two emic items fit this definition, as one is about being preoccupied mentally and physically and putting time to good use, the other is about having an active life using competencies (Items 8 and 10 in Appendix A). Two recent studies (Cieciuch, Schwartz, & Vecchione, 2013; Schwartz et al., 2012) give results of a refined theory of basic individual values, and in this recent model self-direction is divided into two components: One is *autonomy of action* defined as capacity to attain self-chosen goals. The two emic-value items fit the action-component of self-direction.

The other component is *autonomy of thought* defined as developing and using one’s understanding and competence. Three value items are proposed for autonomy of thought (Schwartz et al., 2012, p. 25). These items operationally define this component. They are about creativity, forming own opinions-ideas, and learning for self-improvement. The emic values (Items 5, 18, 20: creativity, capacity use and to be knowledgeable) are analogous to these etic values, and can be considered as self-direction-thought values. But they take place on the joint region; because the PVQ self-direction item used in this study represents the action-component (Item 30 in Appendix A).

On the other hand, emic items for meaning and enthusiasm (Items 11 and 19 in Appendix A) hold up features such as purpose in life, spiritual satisfaction, wisdom and enthusiasm^iv^. These features remind *growth* (Maslow, 1968) or *becoming* concepts (Allport, 1955). When taken together with the above three emic items they seem to be complementary, since knowledge-capacity-creativity (thought) possibly lead the way to meaning. Altogether they signify an attribute of a broader scope. So, restricting ourselves with the results of this study we called this region “intellectualism”. Further research is needed for clarification.

In this study, as far as the motivational continuum assumption is considered, the arrangement of items at the two-dimensional space supports this motivational continuum. The nine value types composed with 10 PVQ and 23 Turkish indigenous items have Cronbach-alpha reliabilities between .574 and .843 (Reliability and mean values are given on Table 4).

### Value Structure

Another important aspect of the theory is the value structure referred as the principle of congruence and conflict. The closer the values are in either direction around the circle, the more similar their motivational goal, the more positive their correlation is. The more distant they are, the more dissimilar they are, the lower or negative their correlation. Table 2 gives the correlations between value types.

**Table 2:** Correlations Between Value Types

	AP	SE	CO	TR	BE	UN	INT	SD	SH
AP	—								
SE	.352**	—							
CO	.232**	.628**	—						
TR	.076	.311**	.573**	—					
BE	.022	.220**	.398**	.312**	—				
UN	-.159**	-.028	.099*	.068	.241**	—			
INT	.178**	.262**	.313**	.128**	.518**	.101*	—		
SD	.192**	.204**	.247**	.073	.403**	.064	.530**	—	
SH	.400**	.204**	.124**	-.050	.123*	.047	.325**	.539**	—

*Note.* AP = Achievement/Power; SE = Security; CO = Conformity; TR = Tradition; BE = Benevolence; UN = Universalism; INT = Intellectualism; SD = Self direction; SH = Stimulation/Hedonism.

*p < .05. **p < .01.

The highest correlations are between values at the same region or the joint region. The correlations decrease with higher distances, but these changes are not so orderly. For instance security has higher correlation with ach/power (*r* = .352) than with tradition (*r* = .311) although security and tradition are of the same higher order value region. Likewise, correlation of benevolence with intellectualism (*r* = .518) and self-direction (*r* = .403) is higher than with universalism (*r* = .241) of the same motivation type. This may be because of the peripheral location of universalism in the dimensional space. In fact, universalism has the lowest correlations with every other value type.

The theory proposes negative relation between opposite poles, because the poles of each dimension indicate different interests: individual interest opposing to collective interest. Table 3 gives the correlations between higher order value types.

**Table 3:** Correlations Between Higher-Level Value Types

	Self- Enhancement	Conservation	Self-Transcendence	Openness to change
Self-Enhancement	—			
Conservation	.247**	—		
Self-Transcendence	-.111**	.211**	—	
Openness to change	.210**	.233**	.279**	—

**p < .01.

The negative relation between self-enhancement (individual interest) and self-transcendence (collective interest) supports this assumption (*r* = -.111). However, openness to change (individual interest) and conservation (collective interest) is in significant positive relation (*r* = .233). From Table 2 it is seen that only tradition has non-significant relations with stimulation/hedonism (*r* = -.050) and with self-direction (*r* = .073) of the opposite pole. It is interesting to note that openness to change values and conformity-security share the highest mean scores, and tradition has the lowest (Table 4).

**Table 4:** Means and Reliabilities of Value Types

	INT	SH	SD	SE	CO	BE	AP	UN	TR
Mean	5.26	5.21	5.14	5.14	5.01	4.83	4.77	4.66	4.19
SD	.61	.81	.71	.83	.80	.76	.93	1.46	1.37
alpha	.767	.722	.574	.591	.735	.648	.843	—^a^	.660

*Note*. INT = Intellectualism; SH = Stimulation/Hedonism; SD = Self direction; SE = Security; CO = Conformity; BE = Benevolence; AP = Achievement/Power; UN = Universalism; TR = Tradition.

^a^Single item value style.

Although conformity and tradition are positively correlated, their correlations with the opposite pole differ (Table 2). This indicates that they are distinct values. While conformity includes interest in close others, tradition signifies interest in one’s culture or religion (Schwartz & Boehnke, 2004). However, just like the other two conservative values, tradition also has significant positive relation with intellectualism, contrary to its non-relation with the other openness to change values. This may be indicating some conditions when social interests are not opposed to individual interests.

On the other hand, conformity and security have positive relations with the values at the opposite pole. Both conformity and security include value items related to family, which points out to the possibility of family as a distinct value region as was previously inferred for the configuration of WAG items in Figure 3.

## Discussion

### Value Arrangement

Turkish value studies done with Schwartz value survey show agreement with the pan-cultural importance of values (Bilsky, Janik, & Schwartz, 2011; Schwartz, 1994; Schwartz & Bardi, 2001; Schwartz & Sagie, 2000). This study, on the other hand is done with value items, 2/3 of which are indigenous. These items are derived from responses given to two questions: “What is the purpose of working?” and “Which achievement of yours you consider as a success?”. The responses are about the desirable end states. However, they are limited with work and achievement; they don’t grasp life as a whole. In spite of this limitation, and although the majority of the items are indigenous, connection between emic and etic items are semantically appropriate and the value arrangement is in agreement with Schwartz value model. The order of values only with 23 emic items (Figure 3) is also in accordance with this model, meaningfully forming the opposite poles of two dimensions.

Addition of *intellectualism* to the model does not violate the theoretical arrangement of values. The closeness of intellectualism to self-direction (*r* = .530) gives evidence for similarity as representatives of intrinsic motivation. However, formation of a distinct region may be pointing either to some cultural complexities in values, or to another type of self-direction or another region under openness to change. The relation of tradition with different types of openness to change values may also be reflecting a difference between self-direction and intellectualism. Traditional values may assimilate values originating from enrichment needs but not those from autonomy, just because autonomy has threatening but enrichment has some safeguarding aspects for the anxiety-based tradition.

### Culture-Specific Concepts

Kağıtçıbaşı (2005) proposes the concept of *autonomous-related self* as a feature of Turkish culture. For Turks autonomy is not seen from an individualistic perspective. There is an intersection between autonomy and relatedness. When the self-direction item is compared with emic value items, a difference in the connotation of autonomy is revealed. Self-direction item implies an autonomous individual who distances self from others (Item 30: “It is important for him/her to make own decisions; s/he likes to manage things as s/he wants”). Emic value items, on the other hand (Items 8 and 10) imply autonomy without denying or disowning relatedness. Especially Item 10 is a perfect example of autonomous-related self where the person is interested in active life and in using competencies together with establishing social relations.

The importance of relatedness is also noticeable with the family notion, which embraces items of different value regions, like family wellbeing and child welfare (security), order in life and trustworthiness (conformity). Repetition of family reminds and possibly gives hints about different cultural variations in responding to values.

Another cultural prototype is seen in a Turkish value study on university students, which demonstrate that one of most important value that is expected from parents to teach their children is *respect* (Erdem Artan et al., 2005). Smith, Türk Smith, and Christopher (2007) also mention respect as a superordinate value for Turks. In their study, it is seen that in describing the *good person* the most frequently used term is *respectful*. Respect is used in a variety of context, and expressions used for respect are various. All these findings point to the fact that value types may be expressed differently within different cultures. These differences may express the same universal value but with a different connotation, which may lead to differences in behavior.

### Deviation From Pan-Cultural Estimations Indicating Further Culture-Specific Concepts

There is some dissimilarity between pan-cultural and indigenous value ratings. Hedonism and stimulation, which are rated as the least important by 56 nations -Turks included- (Schwartz & Bardi, 2001) are the most important in this study. Benevolence and universalism are rated the most important, whereas in this study the same values are rated as less important (6th and 8th) (Table 4). Why is this difference between etic and emic studies? Smith, Türk Smith, and Christopher (in press) answer this question by comparing it with the consensus of Americans and Turks on cuisine. Americans invite a Turkish family to a banquet. Here a variety of dishes representing all major foods are served. Turks are asked to sample among the dishes, and it is seen that Turks prefer dishes that match the American’s favorites. This congruence does not demonstrate “that the same dishes would be the first to come to mind, when Turks planned a meal at home” (p. 12). So, the similarity of ratings does not always mean that the concepts are similarly understood. For instance, in this study 10 of the emic items are derived from a study searching for achievement goal (Table 1). However none of them formed a group specifically with the etic achievement item. Schwartz (1992, 1994; Bilsky et al., 2011; Schwartz & Sagie, 2000) describes achievement as personal success through demonstration of competence according to social standards. The achievement item (Item 24) presents a person expecting admiration when s/he demonstrates abilities. Yet, in the Turkish culture, real feeling of success emerges when one makes the family and close-others *proud* of what s/he did. Giving pride to others is more valuable than receiving admiration from others for achievement. Expression of this kind of achievement item would possibly be rated as a more important value, and would not be united with power.

Another deviation is seen in the relation of opposite poles. Contrary to the principle of conflict between the opposite value poles, this study points to a culture-specific aspect of responding to motivational goals. The results of this study show that for Turkish university students, to be progressive is as important a value as preservative values.

There are other Turkish value studies with discrepant results. For instance in a study it was seen that questioning tradition is as important for Turkish youth as respect for tradition (Konrad Adenauer Foundation, 1999). Another example comes from Turkish teachers who also give discrepant value responses. In the study done with 56-item SVS with additional 4 items, Turkish teachers gave similar responses to autonomy and to in-group attachment (Kuşdil & Kağıtçıbaşı, 2000). Several other studies gave evidence of conflicting values embodied by Turks; like modesty and personal capability, being used equally desirable in self-descriptions (Türk Smith & Tevrüz, 1996).

Rokeach (1973) states that values, especially the main ones, should predict behavior. Empirical studies give behavioral inconsistencies with values (Boyatzis, Murphy, & Wheeler, 2000). However there is evidence of joint structure of values and value expressive behaviors (Bardi & Schwartz, 2003). Having in mind the opposite main values the subjects of this study hold as equally important, one becomes curious about the kind of behaviors to be predicted. An event in Turkey, introduced by the media as *Taksim Gezi Park* protests seems to give some interesting clues about this issue, where on the one hand, all the values of the openness to change, on the other hand the mostly repeated concept family of the conservation pole were on the scene. The protests began without any organizing leadership on May 2013 against the urban development plan for Taksim Gezi Park in Istanbul. It spread to many other cities throughout Turkey, and even to Turkish groups living in various foreign countries. However, events leading to protests were various. For example since 2011 the government became increasingly authoritarian and pan-Islamic. Restrictions on freedom of speech, on press and on the internet-use increased, while the opposition to environmental issues, which seemed to be threatening, was dismissed. What is so insightful, inspirational, and interesting is the humor, entertainment, music, dance, art and wisdom the young protestors put in their protesting behavior, side by side and hand in hand with their families, as if the two opposite values were operating together. Unless empirically investigated, linking these behaviors to the mentioned values is questionable, but it is challenging for inquisitiveness! In order to establish more clear results about value-behavior link, the need for mediating factors is proposed. For example, operating philosophy (Boyatzis et al., 2000), life style (Brunsø, Scholderer, & Grunert, 2004) and mindsets (Torelli & Kaikati, 2009) are some of them. These variables also may have an effect on giving primacy to opposite poles.

### Conclusion

The results of this study support the motivational structure of values, but also results point to some cultural complexities, which should be taken into consideration. WAG scale surely has a Turkish cultural bias. It has culture specific items for culture understanding. For example, Turkish understanding of autonomy does not link values like meaning and enthusiasm to self-direction; conservation-values become meaningful with the concept of family. Emic studies seem to have the potential to expose some unexpected findings. On the other hand, the results of this study would not be as meaningful without a theory based on etic research, but new insights gained by emic studies are as functional. Indigenous studies must be encouraged if new eras of complexities of values and cultures are to be discovered and understood. In fact, there are arguments for making etic and emic approaches complementary, and suggestions about methods to be used (Lu, 2012).

## Appendices

### Appendix A: Value Scale Items

**Table:**

1. Contributing to society^a^	It is important to him/her to be worthwhile for society, to contribute to nation’s development and economy, and serve in shaping a peaceful country. S/He wants to leave a charitable future for the coming generation.
2. Status^a^	It is important for her/him to gain status, authority and power, to have a career and be respectful in society.
3. Livelihood^a^	It is important to her/him to ensure her/his livelihood, guarantee her/his future, to gain money and reach better conditions for living. S/He wants to be able to buy anything s/he wants, and to be a rich person.
4. Family wellbeing^a^	It is important to her/him to ensure family wellbeing and to assure a better life for the offspring.
5. To be knowledgeable^a^	S/He wants to be knowledgeable, to bring out something using own capacities. It is important to her/him to learn new things, to improve self, and get insight about life.
6. Religiousness^a^	It is important to her/him to get god’s consent, to get ready for her/his hereafter and to fulfill religious duties.
7. Avoid missteps^a^	It is important to protect her/himself from alienation and to avoid an immoral life; to be committed to life, to be useful, peaceful and healthy.
8. Being busy^a^	It is important to her/him to have something to do, to be preoccupied mentally and physically, and to put her/his time to good use.
9. Pleasure^a^	S/He wants to satisfy personal pleasures and hobbies, and have fun. S/He gives importance to fulfill her/his dreams.
10. Being active^a^	It is important to her/him to have an active life, to use competencies, build up social relations, and be of help to people.
11. Meaning^a^	It is important to her/him to find a purpose in life, accomplish something notable in life, and reach spiritual satisfaction.
12. Orderly life^a^	S/He wants to set up a proper family, to own a business and have her/his offspring. It is important to her/him to have an orderly life.
13. Performing desired profession^a^	It is important to her/him to perform with enjoyment the profession s/he cares for.
14. Position^a^	It is important to her/him to have a good standard of life and a distinguished position in society. S/He wants to have a good career and get a good position, to work in a distinguished work environment and to have a respectful place in society.
15. Gaining money^a^	S/He wants to gain a lot of money and become economically comfortable and free. It is important to her/him to have a good job and position.
16. Trustworthiness^a^	Being a dependable person for her/his family is important to her/him. S/He wants to be a helpful, honest person who appreciates her/his place.
17. Child welfare^a^	S/He wants to see that her/his children get good education. It is important to her/him to make arrangements for children’s future and witness their achievement.
18. Creativity^a^	It is important to her/him to be cultivated. S/He wants to use in daily life what s/he has learned, to be innovative and carry out things not yet achieved.
19. Enthusiasm^a^	It is important to her/him to be a hardworking person, to use her/his wisdom, and to have enthusiasm in whatever s/he does.
20. Capacity use^a^	S/He wants to use and get beyond her/his capacity, and to achieve what is difficult. It is important to her/him to reach the goals s/he aims.
21. Protect environment^a^	It is important to her/him to be useful for the environment, to be an environmentalist and a pacifist.
22. Integrity^a^	It is important to her/him to have principles and never give up these principles.
23. Set up family^a^	It is important to her/him to get married and to set an order in life.
24. Achievement^b^	It is important to her/him to show her/his abilities. S/He wants people to admire what s/he does.
25. Benevolence^b^	It’s important to her/him to be helpful to people around her/him. S/He works for their goodness.
26. Conformity^b^	It is important to her/him always to show socially appropriate behavior.
27. Hedonism^b^	S/He seeks every chance s/he can to have fun. It is important to her/him to do things that give her/him pleasure.
28. Power^b^	It is important to her/him to be rich. S/He wants to have a lot of money and expensive things.
29. Security^b^	It is important for her/him to avoid things, which might endanger her/his safety.
30. Self-direction^b^	It is important to her/him to make her/his own decisions about what s/he does.
31. Stimulation^b^	It is important to her/him to have an exciting life. S/He likes surprises.
32. Tradition^b^	S/He believes it is best to do things in traditional ways; s/he has respect for tradition.
33. Universalism^b^	S/He thinks it is important that every person in the world be treated equally and has equal opportunities.

^a^From WAG scale. ^b^From PVQ scale.

### Appendix B: Distances

**Table:**

Distances																																	
	1	2	3	4	5	6	7	8	9	10	11	12	13	14	15	16	17	18	19	20
1. contributing_society	0.000																			
2. status	1.448	0.000																		
3. livelihood	1.478	0.076	0.000																	
4. family_wellbeing	1.194	0.474	0.444	0.000																
5. tobe_knowledgeable	0.716	1.283	1.342	1.279	0.000															
6. religiousness	0.888	1.187	1.168	0.728	1.372	0.000														
7. avoid_missteps	0.630	1.049	1.050	0.659	1.051	0.322	0.000													
8. beingbusy	0.961	0.983	1.051	1.096	0.376	1.390	1.079	0.000												
9. pleasure	1.277	0.694	0.770	0.972	0.788	1.472	1.203	0.413	0.000											
10. beingactive	0.978	0.824	0.891	0.948	0.497	1.297	0.998	0.160	0.311	0.000										
11. meaning	0.429	1.143	1.189	1.018	0.367	1.009	0.689	0.533	0.857	0.552	0.000									
12. orderlylife	0.988	0.827	0.805	0.364	1.276	0.364	0.370	1.198	1.194	1.077	0.947	0.000								
13. desired_prof	0.783	1.099	1.161	1.131	0.192	1.314	0.994	0.200	0.602	0.305	0.368	1.171	0.000							
14. position	1.399	0.116	0.192	0.530	1.187	1.214	1.047	0.876	0.578	0.718	1.072	0.863	1.001	0.000						
15. gainmoney	1.437	0.081	0.155	0.530	1.234	1.227	1.071	0.922	0.619	0.764	1.115	0.873	1.047	0.046	0.000					
16. trustworthy	0.560	0.946	0.962	0.636	0.855	0.527	0.211	0.868	1.008	0.791	0.503	0.458	0.787	0.924	0.954	0.000				
17. childwellfare	1.069	0.623	0.601	0.164	1.236	0.567	0.504	1.100	1.038	0.963	0.942	0.205	1.106	0.663	0.670	0.510	0.000			
18. creativity	0.563	1.288	1.342	1.228	0.153	1.251	0.932	0.473	0.868	0.560	0.244	1.186	0.273	1.202	1.247	0.747	1.167	0.000		
19. enthusiasm	0.664	0.829	0.874	0.736	0.547	0.917	0.608	0.473	0.647	0.396	0.315	0.748	0.423	0.761	0.803	0.398	0.690	0.495	0.000	
20. capacity_use	0.665	0.939	0.992	0.898	0.380	1.067	0.750	0.339	0.613	0.315	0.246	0.918	0.254	0.856	0.901	0.540	0.860	0.351	0.171	0.000
21. protect_environment	0.094	1.466	1.500	1.239	0.642	0.974	0.704	0.912	1.248	0.943	0.392	1.054	0.727	1.410	1.450	0.613	1.122	0.490	0.659	0.636
22. integrity	0.387	1.331	1.341	0.974	0.998	0.512	0.322	1.137	1.357	1.100	0.641	0.688	1.002	1.312	1.341	0.389	0.823	0.855	0.712	0.800
23. setup_family	1.066	0.917	0.887	0.444	1.397	0.320	0.436	1.327	1.319	1.206	1.060	0.130	1.297	0.964	0.969	0.562	0.303	1.302	0.874	1.043
24. ACHIEVEMENT	1.544	0.322	0.388	0.771	1.220	1.446	1.258	0.868	0.490	0.724	1.176	1.100	1.028	0.242	0.248	1.115	0.902	1.264	0.883	0.939
25. BENEVOLENCE	0.266	1.185	1.216	0.958	0.606	0.798	0.489	0.761	1.035	0.749	0.247	0.804	0.611	1.134	1.172	0.347	0.849	0.468	0.403	0.435
26. CONFORMITY	0.838	0.828	0.820	0.412	1.118	0.385	0.249	1.059	1.095	0.948	0.784	0.163	1.020	0.842	0.859	0.295	0.255	1.024	0.599	0.765
27. HEDONISM	1.353	0.610	0.686	0.933	0.897	1.482	1.229	0.525	0.116	0.410	0.941	1.184	0.709	0.495	0.532	1.043	1.015	0.970	0.706	0.695
28. POWER	1.748	0.309	0.310	0.751	1.517	1.477	1.356	1.181	0.820	1.030	1.422	1.114	1.327	0.351	0.311	1.255	0.910	1.543	1.112	1.201
29. SECURITY	1.341	0.589	0.537	0.205	1.479	0.738	0.757	1.301	1.162	1.152	1.204	0.400	1.334	0.671	0.659	0.783	0.273	1.422	0.933	1.099
30. SELF_DIRECT	1.117	1.105	1.177	1.269	0.452	1.586	1.274	0.196	0.451	0.322	0.699	1.390	0.334	0.992	1.037	1.064	1.284	0.585	0.669	0.530
31. STIMULATION	1.383	0.820	0.895	1.124	0.830	1.624	1.350	0.459	0.156	0.406	0.956	1.349	0.658	0.704	0.741	1.152	1.193	0.931	0.778	0.720
32. TRADITION	0.801	1.255	1.242	0.809	1.328	0.114	0.297	1.376	1.490	1.295	0.962	0.449	1.285	1.274	1.290	0.507	0.646	1.200	0.905	1.045
33. UNIVERSALISM	0.501	1.738	1.784	1.589	0.596	1.386	1.110	0.963	1.363	1.056	0.595	1.447	0.765	1.663	1.707	0.992	1.490	0.496	0.910	0.811

**Table:**

Distances (continued)														
	21	22	23	24	25	26	27	28	29	30	31	32	33
21. protect_environment	0.000													
22. integrity	0.478	0.000												
23. setup_family	1.138	0.736	0.000											
24. ACHIEVEMENT	1.542	1.502	1.204	0.000										
25. BENEVOLENCE	0.286	0.394	0.902	1.279	0.000									
26. CONFORMITY	0.899	0.569	0.280	1.067	0.643	0.000								
27. HEDONISM	1.331	1.406	1.306	0.380	1.104	1.099	0.000							
28. POWER	1.761	1.640	1.191	0.331	1.483	1.130	0.711	0.000						
29. SECURITY	1.395	1.079	0.420	0.908	1.121	0.513	1.115	0.816	0.000					
30. SELF_DIRECT	1.059	1.322	1.519	0.939	0.937	1.254	0.565	1.266	1.473	0.000				
31. STIMULATION	1.347	1.490	1.475	0.574	1.152	1.249	0.210	0.904	1.312	0.426	0.000			
32. TRADITION	0.890	0.417	0.425	1.500	0.737	0.433	1.508	1.552	0.837	1.571	1.640	0.000		
33. UNIVERSALISM	0.412	0.887	1.539	1.748	0.645	1.287	1.465	2.011	1.761	1.045	1.421	1.301	0.000	
